# A rapid, simple method for the genetic discrimination of intact *Arabidopsis thaliana *mutant seeds using metabolic profiling by direct analysis in real-time mass spectrometry

**DOI:** 10.1186/1746-4811-7-14

**Published:** 2011-06-10

**Authors:** Suk Weon Kim, Hye Jin Kim, Jong Hyun Kim, Yong Kook Kwon, Myung Suk Ahn, Young Pyo Jang, Jang R Liu

**Affiliations:** 1 Biological Resource Center, Korea Research Institute of Bioscience and Biotechnology (KRIBB), 125 Gwahak-ro, Yuseong-gu, Daejeon, 305-806, Korea; 2 Plant Systems Engineering Research Center, Korea Research Institute of Bioscience and Biotechnology (KRIBB), 125 Gwahak-ro, Yuseong-gu, Daejeon, 305-806, Korea; 3 Division of Pharmacognosy, Kyung Hee University, 1 Hoegi dong Dongdaemun-Gu, Seoul, 130-701, Korea

**Keywords:** Arabidopsis thaliana, Direct analysis in real-time mass spectrometry (DART-MS), partial least squares-discriminant analysis (PLS-DA), seed

## Abstract

**Background:**

Efficient high throughput screening systems of useful mutants are prerequisite for study of plant functional genomics and lots of application fields. Advance in such screening tools, thanks to the development of analytic instruments. Direct analysis in real-time (DART)-mass spectrometry (MS) by ionization of complex materials at atmospheric pressure is a rapid, simple, high-resolution analytical technique. Here we describe a rapid, simple method for the genetic discrimination of intact *Arabidopsis thaliana *mutant seeds using metabolic profiling by DART-MS.

**Results:**

To determine whether this DART-MS combined by multivariate analysis can perform genetic discrimination based on global metabolic profiling, intact *Arabidopsis thaliana *mutant seeds were subjected to DART-MS without any sample preparation. Partial least squares-discriminant analysis (PLS-DA) of DART-MS spectral data from intact seeds classified 14 different lines of seeds into two distinct groups: Columbia (Col-0) and Landsberg erecta (Ler) ecotype backgrounds. A hierarchical dendrogram based on partial least squares-discriminant analysis (PLS-DA) subdivided the Col-0 ecotype into two groups: mutant lines harboring defects in the phenylpropanoid biosynthetic pathway and mutants without these defects. These results indicated that metabolic profiling with DART-MS could discriminate intact *Arabidopsis *seeds at least ecotype level and metabolic pathway level within same ecotype.

**Conclusion:**

The described DART-MS combined by multivariate analysis allows for rapid screening and metabolic characterization of lots of *Arabidopsis *mutant seeds without complex metabolic preparation steps. Moreover, potential novel metabolic markers can be detected and used to clarify the genetic relationship between *Arabidopsis *cultivars. Furthermore this technique can be applied to predict the novel gene function of metabolic mutants regardless of morphological phenotypes.

## Background

Functional genomics of higher plants is conducted primarily using a phenotype-based approach. A knockout or over-expressed gene is assumed to produce an overt phenotype in a model plant. However, in practice a large proportion of mutants show no visible morphological phenotype or the phenotype results from a secondary or pleiotropic change, which hinders identification of the gene function. To achieve the practical goal of functional genomics, a more robust characterization system is required to identify mutants.

Global metabolic profiling coupled with statistical analysis is often used for diverse plant biotechnology applications including rapid discrimination between plant species [1-3], differentiation of cultivars or ecotypes [4-6], identification of genetically modified plants [7-13], and metabolic evaluation of commercial food stocks or medicinal herbs [14-18]. These techniques have also been successfully applied to plant functional genomics [19-22]. Recently, plant metabolomics combined with transcriptomics has been used to characterize systemic physiological stress responses [23-26].

Metabolic profiling, which can reveal the metabolic phenotypes of mutants, is most often carried out using gas chromatography (GC)/MS, liquid chromatography (LC)/MS, and proton nuclear magnetic resonance (1H NMR) [27]. For example, Messerli et al. [11] reported that metabolite fingerprinting with GC-MS differentiated *Arabidopsis *mutants defective in starch metabolism from other mutants, indicating that non-targeted metabolic profiling of mutants provides clues about the mutated gene(s). However, it is difficult to achieve high throughput with these instruments primarily due to complicated sample preparation, large sample requirements, and time-consuming operation. Recently, DART-MS has been used for a non-invasive, high-throughput metabolic profiling of samples from various organisms. This new MS technique does not require sample preparation or vacuum ionization, making it an extremely versatile high-throughput system [28-30].

DART ion source is a recently developed ambient ion source which can ionize various organic molecules in diverse samples directly from the surface. In open air conditions, helium, as a carrier gas, produces protonated water clusters from atmospheric water molecules then transfer the proton into molecules in the samples [28]. DART ion source is especially powerful when it is combined with high resolution mass analyzer as it gives exact molecular weight of ionized compounds from the samples and provides matching molecular formula thereof. DART-MS has been adopted in various qualitative analysis of organic molecules including pharmaceuticals, metabolites, synthetic organic molecules and phytochemicals [31-33]. For the quantification purpose, well-known anti-oxidative natural product, curcumin was successfully quantitated from raw material directly [34]. Furthermore, analytical reproducibility of DART-MS was also confirmed using caffeine-d3 with TLC analysis and [35] and olive oil ion analysis [36]. Since labor-taking sample preparation steps can be omitted in DART-MS analysis, high throughput fingerprinting study of natural resources is possible and this feature is one of the most advantageous characteristic of DART ion source in metabolomics approach. And DART ion source ionize moderately polar to highly non-polar compounds [35]. Therefore phenylpropanoids are easily ionized and well-suited to detection.

In this study we attempted to establish high-throughput discrimination with DART-MS to detect intact mutant seeds. To determine whether metabolic profiling with DART-MS can discriminate seeds based on ecotype and altered metabolism, we used 14 different lines of *Arabidopsis *mutant seeds in two different ecotype backgrounds, including previously identified knockout mutants of the phenylpropanoid biosynthetic pathway.

## Results and Discussion

### DART-MS spectra from Arabidopsis seeds

**Figure 1:**
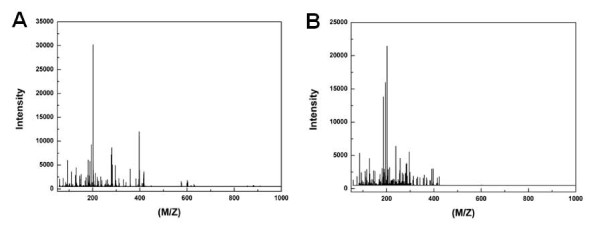
**Representative direct analysis in real-time mass spectrometry (DART-MS) spectra from intact seeds of Col-0 (A) and Ler (B) ecotypes**.

**Figure 2:**
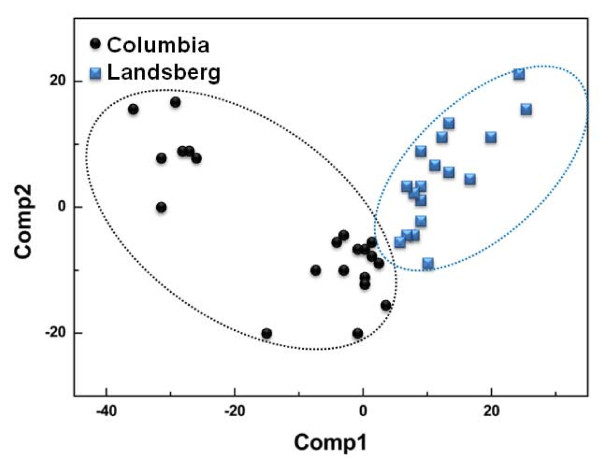
**Partial least square-discriminant analysis (PLS-DA) plot of direct analysis in real-time mass spectrometry (DART-MS) spectral data from intact seeds of two *Arabidopsis *ecotypes (Col-0 and Ler)**. Symbols represent each ecotype. Dotted lines represent Col-0 (black dot circle) and Ler (blue dot circle) backgrounds.

Representative DART-MS spectra from intact seeds of two *Arabidopsis *ecotypes, Col-0 and Ler are shown in Figure 1. More than 319 peaks were detected from intact seed by DART-MS analysis (Additional file 1). Spectral differences between the two ecotypes were significant. Intact seeds of Col-0 ecotypes produced more prominent peaks compared to those of Ler ecotypes. These results implied that there were quantitative and qualitative differences in metabolite patterns between two *Arabidopsis *ecotypes. Interestingly, intact seeds of Col-0 and Ler ecotypes could be successfully discriminated using DART-MS spectral data combined by PLS-DA, even though there is no apparent morphological differences (size, color, or shape) in seed morphology with naked eye (Figure 2). However, principal component analysis (PCA) could not fully differentiate these groups (Additional file 2). In cross-validation, each case was classified by the functions derived from all other cases; 100% of original grouped cases and 100% of cross-validated grouped cases were correctly classified (Table 1). These results indicated that metabolic profiling with DART-MS spectral data could discriminate two *Arabidopsis *ecotypes even though there were no visible, distinctive characteristics in the seed morphology.

**Table 1:** Cross validation between two *Arabidopsis *ecotypes from direct analysis in real-time mass spectrometry (DART-MS) spectral data of intact seeds

Intact seed		Prediction		Total
			
		Col-0	Ler	
Cross-validated count	Col-0	21	0	21

	Ler	0	21	21

Total		21	21	42

The discriminant functions were the first five discriminant components from PLS-DA analyses (5 data). In cross-validation, each case was classified by the functions derived from all other cases; 100% of original grouped cases and 100% of cross validated grouped cases were correctly classified.

### Genetic discrimination of two ecotype backgrounds from DART-MS spectral data of intact seeds

Identification of gene function is limited when based on mutant morphological phenotype, which is often silent or the result of a secondary or pleiotropic changes. To overcome this limitation, non-targeted metabolic profiling of the mutant may provide clues about the mutated gene(s). To investigate the discrimination possibility between genetic defective mutants from same background, we applied three *Aabidopsis *mutants (*co, ft *and *tt2*) from Landsberg background and nine mutants (*chs, f3h, f3'h, dfr, ldox, ban, pap1-D, phyB*, and *ugt78d2*) from Col-0 in this study.

*co *and *ft *are representative *Aabidopsis *mutants showing late flowering phenotype that is null mutants of CONSTANS (CO) and FLOWERING LOCUS T (FLC), respectively [37]. *tt2 *is the null mutant of TRANSPARENT TESTA 2 (TT2), that is one of R2R3 MYB domain transcription factor and acts as a key determinant in the proanthocyanidin accumulation of developing seed. It is composed of ternary complex with TT8 and TTG1 for correct expression of BANYULS (BAN) in seed endothelium [38-40]. Therefore the seed color of *tt2 *mutant is yellow due to the defectiveness of condensed tannin accumulation in the seed endothelium.

**Figure 3:**
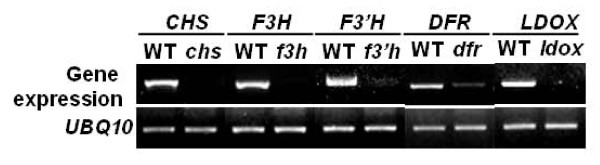
**RNA expression level in each T-DNA inserted mutant lines**. Total RNA were extracted from seven-day old grown seedlings. Gene expression level of each mutant line was analyzed with primers for full length ORF by RT-PCR.

Nnine Arabidopsis mutants (*phyB, pap1-D, chs, f3h, f3'h, dfr, ldox, ban*, and *ugt78d2*) are belonging to Col-0 background. *phyB *and *pap1-D *are a null mutant of phytochrome B (PHYB), primary red/far-red photoreceptor in light signaling transduction pathway and an activated mutant of PRODUCTION OF ANTHOCYANIN PIGMENT 1 (PAP1) act as an activator of phenylpropanoid biosynthesis, respectively [41,42]. The rest of mutants belonging to Col-0 background are seven T-DNA inserted knock-out mutants (*chs, f3h, f3'h, dfr, ldox, ban*, and *ugt78d2*) involved in anthocyanin biosynthesis pathway. The gene expression levels of these mutants (*chs, f3h, f3'h, dfr *and *ldox*) were analyzed by RT-PCR using primer pairs for pull length ORF (Figure 3). This result indicated that all of the mutants were completely defective mutants. But DFR was slightly expressed in *dfr *mutant line, probably resulting from T-DNA insertion on the 3' UTR region of DFR gene. No expression of BAN was observed in both WT and *ban *mutant seedlings as previous reported by Lee et al. [43]. Three mutants could easily be discriminated by the seed color itself. The color of seed coat from *tt2 *and *chs *mutants was yellow, whereas that of *pap1-D *mutant was darker than wild type. Although rest of mutants used in this study has slightly difference in seed color, it could not easily be discriminated without direct comparison with wild type. Furthermore, determination of genetic background from *tt2*, *chs *and *pap1-D *mutants was impossible by naked eye, even though they had an obvious seed color.

**Figure 4:**
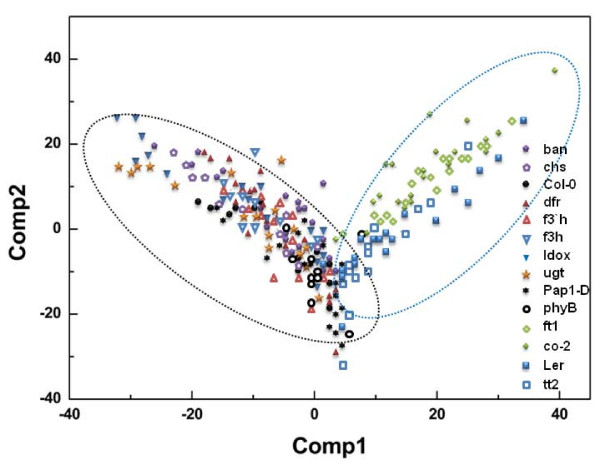
**Partial least square-discriminant analysis (PLS-DA) plot of direct analysis in real-time mass spectrometry (DART-MS) spectral data from intact seeds of 14 *Arabidopsis *mutant lines**. Symbols represent each ecotype and mutant line. Dotted lines represent Col-0 (black dot circle) and Ler (blue dot circle) backgrounds.

PLS-DA of DART-MS spectral data from intact seeds divided the 14 lines of seeds into two distinct groups: Col-0 and Ler ecotype backgrounds (Figure 4). In cross-validation, each case was classified by the functions derived from all other cases; 100% of original grouped cases were correctly identified, whereas 80.6% of cross-validated grouped cases were correctly classified (Table 2). In particular, the mutant seed lines *chs, co*, and *ft *were perfectly predicted, whereas *dfr *was incorrectly classified. What *dfr *shown comparatively low level of accuracy in cross-validation thought to be partial production of metabolic components caused by slightly expressed DFR in the mutant. In contrast, few prediction errors were made at the ecotype level by analysis of intact seeds. All mutant lines (*chs, f3h, f3'h, dfr, ldox, ban, pap1-D, phyB*, and *ugt78d2*) and wild type seeds belonging to the Col-0 ecotype were correctly predicted within the Col-0 ecotype. Likewise, mutant lines (*co *and *ft*) and wild type Ler seeds were correctly predicted within the Ler ecotype. However, prediction of the *tt2 *line was 90.5% accurate. These results indicated that DART-MS spectrometry combined with multivariate analysis of intact seeds was able to discriminate the lines of seeds at least the ecotype level.

**Table 2:** Cross validation of *Arabidopsis *ecotypes from direct analysis in real-time mass spectrometry (DART-MS) spectral data of intact seed

	Prediction														
	
Cross-validated count	ban	chs	co	Col-0	dfr	f3'h	f3h	ft	ldox	Ler	pap1-D	phyB	tt2	ugt78D2	Total
ban	18	0	0	0	1	1	0	0	0	0	0	1	0	0	21
chs	0	21	0	0	0	0	0	0	0	0	0	0	0	0	21
co	0	0	21	0	0	0	0	0	0	0	0	0	0	0	21
Col-0	0	0	0	19	0	0	0	0	0	0	0	2	0	0	21
dfr	2	3	0	0	8	1	0	0	2	0	4	1	0	0	21
f3'h	2	4	0	1	0	13	1	0	0	0	0	0	0	0	21
f3h	1	2	0	0	0	0	17	0	0	0	0	1	0	0	21
ft	0	0	0	0	0	0	0	21	0	0	0	0	0	0	21
ldox	1	2	0	0	0	2	3	0	12	0	1	0	0	0	21
Ler	0	0	0	0	0	0	0	0	0	17	0	0	4	0	21
pap1-D	0	0	0	0	0	0	0	0	0	0	19	0	0	2	21
PhyB	1	0	0	0	0	0	0	0	0	0	1	19	0	0	21
tt2	1	0	0	0	0	0	0	0	0	0	1	0	19	0	21
ugt78d2	0	1	0	0	0	1	0	0	0	0	2	4	0	13	21

Total	26	33	21	20	9	18	21	21	14	17	28	28	23	15	294

The discriminant functions were determined using the first five discriminant components determined by PLS-DA analyses (5 data). In cross-validation, each case is classified by the functions derived from all other cases; 100% of original grouped cases, while 80.61% of cross validated grouped cases were correctly classified.

In the present study, we described a robust method for high throughput profiling with DART-MS. The functional identification of genes of mutant *Arabidopsis *is currently based on morphological phenotype. However, up to 85% of the mutants exhibit no overt phenotype [21]. Metabolic alterations may be present in a large portion of these mutants; however, current methods for metabolic profiling are complicated and time-consuming, which precludes high-throughput screening of mutants. In the present study, a robust screening of mutants with altered metabolism was devised with DART-MS using intact *Arabidopsis *mutant seeds. This approach is not limited to screening mutants lacking genes expressed in the seed coat, but may be extended to identification of genes expressed in other seed parts. DART-MS has been used to identify plant compounds [29,30], but this rapid, simple instrumentation has not yet been utilized for high-throughput screening of mutants. Considering the overall PLS-DA results, intact *Arabidopsis *seeds could allow genetic discrimination of ecotypes and sorting of specific mutants harboring defects in the phenylpropanoid biosynthetic pathway (Figures 2 and 4) and flowering time genes.

### Hierarchical clustering of Arabidopsis seeds based on multivariate analysis of metabolic profiling

**Figure 5:**
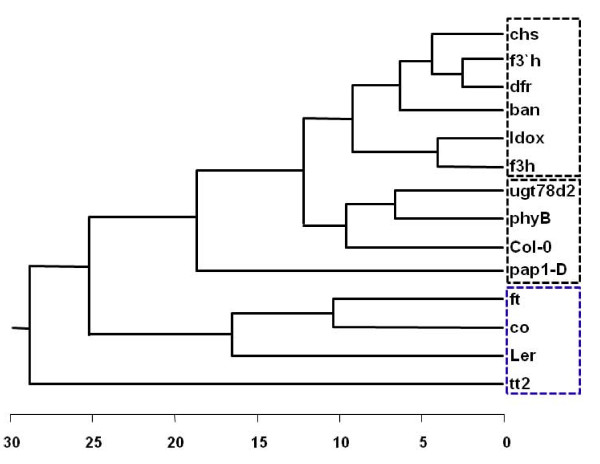
**Hierarchical dendrogram of partial least square-discriminant analysis (PLS-DA) score data from direct analysis in real-time mass spectrometry (DART-MS) spectra of intact seeds**. Rectangles represent Col-0 (black dot square) and Ler (blue dot square) ecotypes.

A hierarchical dendrogram based on PLS-DA of DART-MS spectral data from intact seeds showed that 13 lines (excluding the *tt2 *mutant line) were divided into two major branches by ecotype (Figure 5). Interestingly, the hierarchical dendrogram from intact seed subdivided the Col-0 ecotypes into two subgroups: seven mutant lines lacking a gene involved in the phenylpropanoid biosynthetic pathway except the *ugt78d2 *clustered together, separate from the other three lines (*phyB, pap1-D*, and *Col-0*) (Figure 5).

Anthocyanins are water-soluble vacuolar pigments and belong to a parent class of flavonoids synthesized via the phenylpropanoid pathway. Anthocyanins are found in all tissues of higher plants, and especially proanthocyanidins in the seed coat. UGT78D2 that catalyzes the glucosylation of both flavonols and anthocyanidins which converted to anthocyanins, is highly expressed in anthocyanin-accumulating seedlings, but repressed in condensed tannin-accumulating seed coats [43]. It seems that metabolic flux for the metabolic end-products in mature seed coats is not affected even if UGT78D2 is abolished in *Arabidopsis *seedlings or seeds. It indicated that there might be no differences in metabolic components between WT and *ugt78d2 *mutant seed coats. BAN encodes a core enzyme, anthocyanin reductase of flavonoid biosynthesis. It is convert anthocyanindins to flavan-3-ol, which condensed to colorless proanthocyanins [44]. They are placed only in the seed coat, and confer a brown color to mature seed after oxidation. BAN, also act as a negative regulator of flavonoid biosynthesis during early embryogenesis, and highly expressed in the tannin-accumulating mature seed in Arabidopsis [43,45-47]. Therefore, accumulation of proanthocyanins probably is inhibited in the developing seed coats of *ban *mutants. From this point of view, it is reasonable that *ugt78d2 *grouped with WT, and *ban *clustered with *chs, dfr *and *f3'h*, respectively. Therefore, we inferred that the combination of multivariate analysis and DART-MS, might reflect gene functional relationship on flavonoid biosynthetic pathway such as *ugt78d2 *and *ban *clustered with other associated mutants reasonably.

A hierarchical dendrogram based on PLS-DA of DART-MS spectral data from intact seeds from Ler and three mutant lines (*co, ft *and *tt2*), they were separated into other major branches from Col-0 ecotypes except for the *tt2 *mutant line (Figure 5). TT2 functions as a regulator in proanthocyanidin accumulation in developing seed only when TTG1 is expressed. The ternary complex of TT2, TT8 and TTG1 positively regulate BAN expression in whole seed coats by directly regulating *BAN *promoter activity in plants [39,40,48]. No detection of *BAN *transcript in *tt2, tt8 *and *ttg1 *confer homogeneously yellow hue on their seed coats [39,47]. Therefore, we expected that *tt2 *mutant was clustered with other *tt *mutants in the fravonoid biosynthetic pathway, especially *ban *mutant, regardless of background properties. However, *tt2 *was placed near to Ler branch from the hierarchical dendrogram even though *tt2 *was not included into Ler branch (Figure 5). These result implied that overall metabolic differences between Arabidopsis ecotypes was greater than that of a specific gene, for example metabolic change in tannin accumulation of *tt2 *mutant. Therefore we suggested that there were common metabolic compounds that mainly affected for the ecotype discrimination of Arabidopsis. Considering the overall hierarchical clustering analysis (HCA) results, we concluded that DART-MS spectrometry combined with multivariate analysis of intact seed could not only discriminate *Arabidopsis *seeds at the ecotype level, but could also cluster metabolic genes related to same metabolic pathway. Therefore, we suggest that DART-MS spectrometry may be useful as a tool for rapid discrimination of ecotypes and metabolic mutants of *Arabidopsis*.

### Assignments of chemical compounds for ecotype discrimination

**Figure 6:**
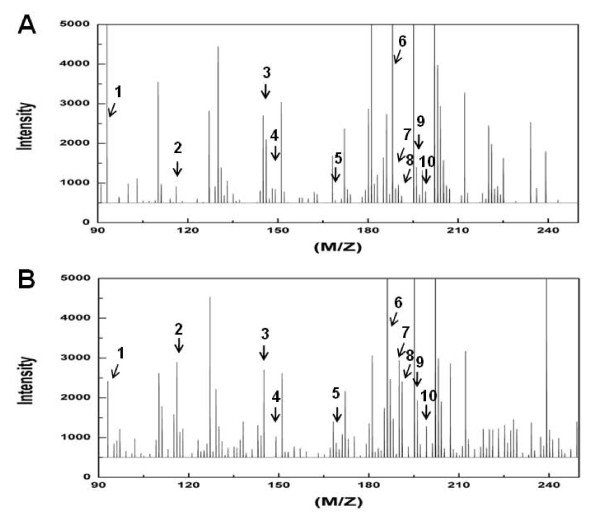
**Enlarged view of direct analysis in real-time mass spectrometry (DART-MS) spectra from intact seeds of Col-0 (A) and Ler (B) ecotypes**. Arrows and numbers represent 10 major compounds for metabolic discrimination between the ecotypes.

Mass spectrometry analysis is one of the most powerful analytical methods available for exact structural identification of organic compounds. In this study, more than 319 peaks were detected from intact seed by DART-MS analysis (Figure 1). These peaks (m/z) have not fully assigned as chemical compound yet, because of lack of Arabidopsis chemical DB. We selected the top 10 most significant metabolites for discrimination between Col-0 and Ler ecotypes from the MS spectral data of intact seeds by logistic regression (Figure 6).

In general, DART ion source commonly produces a mass spectrum consisting of the [M+H]^+ ^molecular cation by proton transfer mechanism. But molecular ion peak of M+ also found commonly by penning reaction. Thus, the ion peak of selected compounds in this study was assigned as molecular ion by penning reaction. However, DART-MS cannot discriminate the same chemical formula compounds similar to all other mass spectrometers. Therefore, with the works of intensive in silico informatics on previous phytochemical studies on Arabidopsis thaliana only gives the information on exact molecular weights for the compounds in samples. Assignment of the 10 metabolites was performed by direct comparison with the online chemical database Plant Metabolic Network (http://www.plantcyc.org) (Table 3).

**Table 3:** Ten major metabolites used for ecotype discrimination from intact seed of *Arabidopsis *ecotypes

No	Compound	***m/z***^a^	Ratio(Col-0/Ler)
1	Glycerol	92.115	2.465

2	L-delta1-Pyrroline-5-carboxylate	114.104	0.466

3	Dimethyl fumarate	145.106	3.526

4	L-Glutamic acid	148.107	0.665

5	Pyridoxal	168.158	0.651

6	NA^b^	185.155	0.236

7	NA^b^	186.170	0.378

8	1-Methoxyindole 3-carboxylic acid methyl ester	190.191	0.351

9	5-Hydroxyconiferyl aldehyde	195.163	0.608

10	5-Hydroxyconiferyl alcohol	197.124	1.844

^a ^mass-to-charge ratio. ^b^Not assigned for pure compound.

The intensity of the molecular ion peak of glycerol (MW = 92.115) was 2.5 times higher on the seed surface of the Col-0 ecotype than that of Ler. The intensity of dimethyl fumarate (MW = 145.106) was 3.5 times higher in seed surface of the Col-0 ecotype than that of Ler. Whereas molecular ions of L-glutamic acid, pyridoxal and 5-hydroxyconiferyl aldehyde was more abundant in Ler than in the Col-0 ecotype. Ward et al. [4] reported that nine *Arabidopsis *ecotypes could be discriminated based on glucose and fumaric acid content by ^1^H NMR spectroscopy. Direct comparison of key metabolites between the report of Ward et al. [4] and this study were not suitable because of difference in organic solvent and plant materials for metabolite extraction steps. In the present study, we conducted direct MS analysis of seed surface without any organic solvent extraction steps. However dimethyl fumarate was the one of key metabolite in seed coat of Col-0 ecotype. We have not fully understood the biochemical and metabolic pathway of fumaric acid and dimethyl fumarate, especially in seed coat yet. If fumaric acid could be actively modified by methyl group in seed coat, the report of Ward et al. [4] and our study showed that fumaric acid derivatives were key metabolites for ecotype discrimination in *Arabidopsis*. In addition, we also found that glycerol had a key role in ecotype discrimination of *Arabidopsis*. Therefore, the report of this study could be applied for the study of glycerol biosynthesis pathway in *Arabidopsis *ecotypes.

Although other MS with higher analytical resolution such as FT-ICR MS can be used for this study, they are suitable for the analysis of unknown species owing to its ultra high mass resolution and accurate mass capacity [49]. AccuTOF, the analyzer used in this study, is an orthogonal acceleration time-of-flight mass spectrometer (oa-TOF-MS) incorporating a single stage reflectron. The resolving power of this analyzer is excess of mass 6,000 (FWHM definition) [50]. Taking into account the relatively high resolution, and fast scan speed with a wide dynamic range, AccuTOF-MS is a powerful tool for high-throughput profiling or chemical fingerprinting of intact seed samples.

## Conclusions

In this study, we demonstrate that DART-MS combined by multivariate analysis allows for rapid screening and metabolic characterization of lots of *Arabidopsis *mutant seeds without complex metabolic preparation steps. Our results represent that mutant lines including wild types were classified to two distinct groups, Col-0 and Ler ecotype backgrounds on PLS-DA from DART-MS spectral data of 14 lines of intact Arabidopsis seed. Furthermore, mutants which are Col-0 background were subdivided into two groups in the hierarchical dendrogram based on PLS-DA, in which one group of defective mutants is related to the phenylpropanoid biosynthetic pathway. These results demonstrate that DART-MS combined by multivariate analysis can discriminate mutants based on quantitative and qualitative differences affecting global metabolic profiles. Considering these results we infer that metabolic profiling with DART-MS could discriminate intact *Arabidopsis *seeds at least ecotype level or metabolic pathway level within same ecotype. Screening mutants in the form of seeds saves the labor time required to grow plants. Thus, we suggest that DART-MS spectrometry combined by multivariate analysis is a useful tool for only rapid screening of metabolic mutants, but also discrimination of ecotypes of *Arabidopsis*. Furthermore plant functional genomics can be carried out based on metabolic profiling of intact *Arabidopsis *mutant seeds by DART-MS in a high throughput manner.

## Materials and methods

### Plant materials

Arabidopsis plants were grown in a growth room at 20 to 24°C under a long day condition (16/8 hr photoperiod at 80 umol m^-2^s^-1^) with white light in the Arasystem (LEHLE SEEDS. USA). All of the seeds were harvested and desiccated for 2 weeks at 30°C chamber for this study. Col-0 and Ler ecotypes were used as wild type of Arabidopsis thaliana. We applied three mutants as the mutant of Ler ecotype background; *co *(*co-2*, cs175), *ft *(f*t-1*, cs56), and *tt2 *(*tt2-1*, cs83), and nine mutants as that of Col-0 ecotype background; *phyB *(*phyB-9, cs6217) pap1-D (cs3884)*, *chs (*salk_064816*), f3h (*salk_113904)*, f3'h (*salk_124157*), dfr (*cs115210*), ldox (*salk_073183*), ban (*salk_040250*) *and *ugt*78d2 (*ugt78d2-1*, salk_049338, [41]). These mutants except T-DNA insertion lines (SALK lines) were gained from the Arabidopsis Biological Resource Stock Center [51]. T-DNA inserted mutants (*chs, f3h, f3'h, dfr, ldox, ban *and *ugt78d2)*, also derived from ABRC were kindly provided by Dr. Giltsu Choi (KAIST, Daejeon, Korea), in which T-DNA inserted position were previously confirmed on the genome of each mutants by PCR method using each genes primer and LBa1 primer for the T-DNA vector, pROK2 (http://signal.salk.edu/), and then selected homozygous T-DNA inserted mutants. To confirm the RNA expression of T-DNA inserted lines, we extracted total RNA from the seven-day-old white-light grown seedling with RNeasy Miniprep Kit (Qiagen, Germany), and reverse-transcribed with M-MLV reverse transcriptase and Oligo(dT)_15_, according to the manufacturer's protocol (Enzynomics, KOREA). They were equalized by ubiquitin (UBQ10; AT4G05320, 5'-AGTCCACACTTCACTTGGTC-3', 5'-TTAGAAACCACC ACCGA-3'). Primers for expression analysis included: CHS (At5g13930, 5'-TCCCCCGGGC ATGGTGATGGCTGGTGCTTCTT-3', 5'-CCGCTCGAGGAGAGGAACGCTGTGCAAGAC G-3'), F3H (At3g51240, 5'-TCCCCCGGGCATGGCTCCAGGAACTTTGACTG-3', 5'-CGGGATCCGAAGCGAAGATTTGGTCGACAG GC-3'), F3'H (At5g07990, 5'- TCCCCCGGGCATGGCAACTCTATTTCTCACAATC-3', 5'-CCGCTCGAGACCCGACCCGAGTCCATAAACG-3'), DFR (At5g42800, 5'-TCCCCCGGGCATGGTTAGTCAGAAAGAGACC-3', 5'CGGGATCCGAGGCACACATCTGTTGTGCTAGC-3'), LDOX (At4g22880, 5'-TCCCCCGGGCATGGTTGCGGTTGAAAGAGTTGA-3', 5'-CGGGATCCGAATCATTTTTCTCGGATACCAATTC-3'), BAN (AT1G61720, 5'-TCCCCCGGGCATGGACCAGCTCTTACACACAC-3', 5'-CCGCTCGAGT TTAGCTTTGA TCAATCCTTT TGA-3')

### DART-MS

A Jeol DART-MS instrument (Tokyo, Japan) was used, which comprised a DART ion source and a JMS-T100TD (AccuTOF) atmospheric pressure ionization time-of-flight mass spectrometer. For positive ion detection, the atmospheric pressure interface potentials were set to the following values: orifice 1 = 10 V, ring lens and orifice 2 = 5 V. The ion guide potential and detector voltage were set to 500 V and 2400 V, respectively. DART electrode potentials were set to needle electrode = 3000 V, electrode 1 = 100 V, electrode 2 = 100 V. Gas temperature was set to 250°C, and the helium gas flow rate was 3 L/min. Each seed was positioned midway between the DART source and mass spectrometer for measurement. Three measurements for each seed were averaged, and three different seeds of each wild type and mutant line were used as replicates.

### Data processing and multivariate statistical analysis

To minimize the influence of sample size, DART-MS spectral data were normalized to total ion count percent. Small noise peaks with low ion intensity value (< 100) were removed from the original spectral data. Multivariate statistical analysis was performed using mean-centered and auto scaled data. These preprocessed metabolomic datasets were imported into R programs for PCA, PLS-DA and HCA. PCA, an unsupervised clustering method, was performed to statistically analyze comprehensive information contained in a data set [52]. Also PLS-DA, a representative supervised data mining algorithm, could give more precise group separation. To create the PLS-DA model, the entire data set is divided into two parts: a training set that was used to build a model, and a test set that was not used in the classification model, but was used to verify the model's predictive ability. To estimate the predictive power and significance of a latent variable of a model, cross-validation was used. Permutation testing also evaluates the statistical significance of the estimated predictive power of a model. After predictive validation by means of cross-validation and response permutation testing, external validation, a more demanding and rigorous mechanism for testing predictive performance consisted of computing predictions for an independent set of test observations (test set). Also, HCA was performed to statistically analyze comprehensive relationship contained in PLS-DA score data from each sample.

To identify the significant metabolites for ecotype discrimination from MS spectral data, P-values for all metabolites were calculated by logistic regression, and 10 metabolites with high P-values were selected. Assignment of 10 metabolites was conducted by comparison with the database of the Plant Metabolic Network (http://www.plantcyc.org).

## List of abbreviations

ASCII: American standard code for information interchange; Col-0: Colombia; DART-MS: Direct analysis in real-time mass spectrometry; HCA: hierarchical clustering analysis; Ler: Landsberg erecta; PC: Principal component; PCA: Principal component analysis; PLS-DA: partial least squares-discriminant analysis.

## Competing interests

The authors declare that they have no competing interests.

## Authors' contributions

JRL conceived and directed this study, and drafted the manuscript. SWK and JHK performed most experimental works and wrote the paper with input from co-authors. HJK and YPJ carried out the DART-MS and identified chemical compounds. YKK carried out the statistical analysis. MSA performed Arabidopsis seed management and contributed to the manuscript. All authors read and approved the final manuscript.

## Supplementary Material

Additional file 1 **Raw data of DART-MS spectra from intact 14 Arabidopsis mutant seeds**. MS spectral data was consisted of three replicates from each seed line. Click here for file

Additional file 2 **PCA score plot of DART-MS spectra from two Arabidopsis ecotypes (Col-0 and Landsberg)**. Click here for file
