# Supplementary figures and images for: A rapid, simple method for the genetic discrimination of intact Arabidopsis thaliana mutant seeds using metabolic profiling by direct analysis in real-time mass spectrometry
Source: Plant Methods. 2011 Jun 10;7:14. doi: 10.1186/1746-4811-7-14 (PMC3138417; doi:10.1186/1746-4811-7-14)

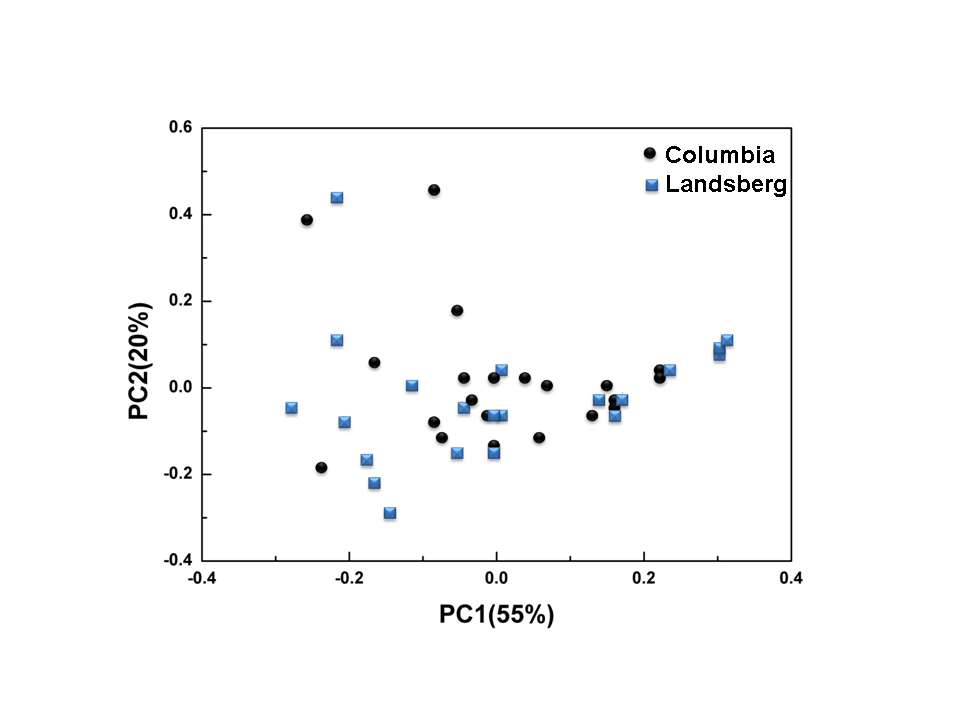

Supplement: Additional file 2 — PCA score plot of DART-MS spectra from two Arabidopsis ecotypes (Col-0 and Landsberg). [file 1746-4811-7-14-S2.TIFF]
